# Application of Levi’s Muscle Index in frailty assessment: comparison of bioimpedance measures among older adults

**DOI:** 10.3389/fmed.2025.1525569

**Published:** 2025-06-26

**Authors:** Kworweinski Lafontant, David H. Fukuda, Estefania Zamarripa, Abigail L. Tice, Jethro Raphael M. Suarez, Chitra Banarjee, Dahee Kim, Jeffrey R. Stout, Joon-Hyuk Park, Rui Xie, Ladda Thiamwong

**Affiliations:** ^1^College of Health Professions and Sciences, Institute of Exercise Physiology and Rehabilitation Science, University of Central Florida, Orlando, FL, United States; ^2^EMBRACE Lab, College of Nursing, University of Central Florida, Orlando, FL, United States; ^3^Wearable Engineering and Assistive Robotics Lab, Department of Mechanical and Aerospace Engineering, University of Central Florida, Orlando, FL, United States; ^4^College of Medicine, University of Central Florida, Orlando, FL, United States; ^5^Department of Disability, Aging, and Technology Cluster, University of Central Florida, Orlando, FL, United States; ^6^Department of Statistics and Data Science, College of Sciences, University of Central Florida, Orlando, FL, United States

**Keywords:** muscle quality, BIVA, phase angle, aging, fitness assessment

## Abstract

**Introduction:**

Frailty is prevalent among older adults and is characterized by reductions in physical function and muscle quality. Despite the emerging clinical utility of bioelectrical impedance analysis (BIA) and phase angle (PhA) as a bioimpedance index, little is known about how bioimpedance indices such as Levi’s Muscle Index (LMI), reactance/height (Xc/Height), and resistance/height (R/Height), relate to physical function and frailty.

**Methods:**

This cross-sectional study examined 208 community-dwelling older adults (female, *n* = 183; age = 74.2 ± 6.9 years; BMI = 30.4 ± 6.4 kg/m^2^) to compare physical function measures and bioimpedance indices across frailty categories determined by the FRAIL questionnaire. PhA, LMI, Xc/Height, and R/Height were all assessed at 50 kHz using a direct segmental multi-frequency InBody s10 BIA device. Physical function was assessed using handgrip strength, postural sway, Timed-Up-and-Go, and the Short Physical Performance Battery. Data were analyzed using Spearman rho (ρ) and Pearson r correlation coefficients, and group differences were examined using Kruskal-Wallis H tests and one-way ANOVA.

**Results:**

PhA (*r* = −0.18, *p* = 0.01) and Xc/Height (*r* = −0.24, *p* < 0.001) were significantly associated with FRAIL scores. LMI and PhA were well correlated with each other (ρ = 0.76, *p* < 0.001), yet Xc/Height was the only bioimpedance index to significantly differ between frailty categories (*F* = 6.39, *p* = 0.002, ηp^2^ = 0.06).

**Conclusion:**

Results suggest Xc/Height may be the only bioimpedance index indicative of frailty among older adults. Given the variety of assessments used to categorize frailty, these conclusions may be limited to the use of the FRAIL questionnaire; future research should compare LMI and PhA using multiple frailty indices.

## 1 Introduction

Physical function naturally declines with aging and contributes to a decrease in skeletal muscle quality (1). While physical function can be readily assessed with measures such as postural sway (PS), the Short Physical Performance Battery (SPPB), the Timed-Up-and-Go (TUG), Sit-to-Stand (STS), and handgrip strength (HGS), these assessments take time to complete and often require qualified supervision to reduce injury risk. Physical function is commonly used as a surrogate measure of frailty, making it a clinically relevant variable for older adult populations (2–4). To promote regular monitoring among older adults, research has focused on identifying predictors of physical function through quick and easy assessments such as bioelectrical impedance analysis (BIA) (5–7).

**FIGURE 1:**
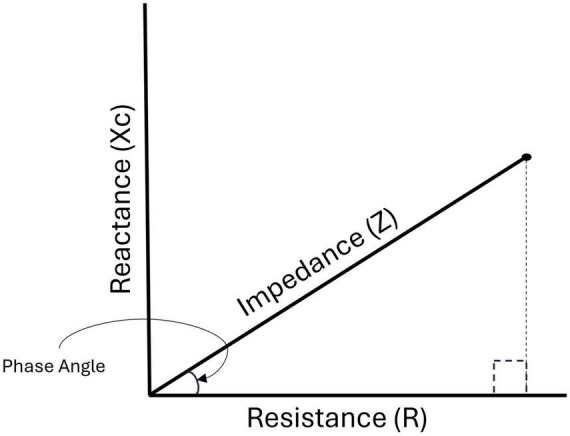
The relationship between impedance, resistance, reactance, and phase angle.

BIA assesses the bioelectrical properties of cells by sending electrical currents through the body and measuring the overall opposition, or impedance (Z), that the current faces (8). Z largely occurs at cell membranes and is comprised of reactance (Xc) and resistance (R), as shown in Figure 1 (8). Xc represents the capacitive properties of cell membranes, while R reflects the resistive properties of cell membranes due to fluid distribution (8). Z, Xc, and R can be used to calculate phase angle (PhA), which is a global indicator of cellular health (9, 10). PhA has demonstrated clinical utility as a predictor of sarcopenia and a risk factor for frailty among older adults (11, 12), suggesting its relationship to muscle quality. Previous research has also demonstrated reduced physical function among older adults with a low PhA (5–7). Beyond PhA, Xc, and R are typically standardized by height to create reactance (Xc/Height) and resistance (R/Height) indices through a technique known as bioelectrical impedance vector analysis. Like PhA, these indices are used to assess cellular health among older adults without predictive equations (13, 14). While the utility of PhA, Xc/Height, and R/Height in this population is well established, there is a lack of research on a recently developed bioelectrical variable, Levi’s Muscle Index (LMI), and its potential as a predictor of physical function among older adults.

LMI was created to evaluate skeletal muscle quality and distinguish training status among athletes without predictive equations (15), suggesting its potential as an objective assessment of frailty. Because predictive equations are not used within LMI (i.e., it is not based on a regression equation), it can be applied to any population given its use of directly assessed bioelectrical properties. Calculated as [(PhA × Height)/R], LMI has been studied in expeditioners, bodybuilding, track and field, and soccer athletes (15–18). Among elite-, high-, and medium-performing soccer athletes, LMI significantly differed (*p* < 0.001), potentially demonstrating an ability to detect physiological adaptations related to performance levels (15). Among track and field athletes, LMI demonstrated weak-to-moderate correlations with aspects of athletic performance, such as velocity and force production during squat jumps, countermovement jumps, and sprints (17). Previous research using LMI among bodybuilders did not compare LMI values between groups (16). Despite its previous research among athletes, to the best of our knowledge, no study has applied LMI among older adults. BIA assessments are useful within the context of both athletics and older adults as a method of estimating body composition and hydration status, as well as assessing global cellular health via PhA, R/Height, and Xc/Height (9, 10, 12). However, with LMI being a recently established bioimpedance index, there is a need to examine its application among older adults specifically.

While the relationship between skeletal muscle quality and physical function among older adults has been previously evidenced (1, 19, 20), there is a lack of research assessing the relationship between LMI and physical function. The foundational study that introduced LMI only reported associations with body cell mass and PhA (15), two measures derived from the same raw bioelectrical variables as LMI. Furthermore, the rationale for the formulation of LMI was that dividing PhA by R/Height would offer an adjustment for variations in hydration status (17). However, PhA already accounts for hydration status using R, making it unclear how LMI improves upon bioimpedance assessments of muscle quality via PhA. Although PhA is currently used as a clinical indicator of physical function and frailty among older adults, LMI may be an innovative metric for clinicians and researchers if it can provide an improved indication. Therefore, the purpose of this study was to assess the relationships between LMI, PhA, Xc/Height, R/Height, physical function, and frailty among older adults. We hypothesized that all bioimpedance indices, including LMI, would be associated with physical function and frailty.

## 2 Materials and methods

### 2.1 Participants

This cross-sectional preliminary investigation was part of a larger study funded by the National Institute on Minority Health and Health Disparities (R01MD018025) and pre-registered on ClinicalTrials.gov (NCT05778604). The study protocol was approved by the University of Central Florida Institutional Review Board (STUDY00003206), conducted in accordance with the Declaration of Helsinki, and previously published elsewhere (21). All participants provided written informed consent prior to participation. A total of 274 participants were recruited from the greater Orlando, FL, metropolitan region via fliers at community centers and events such as health fairs, local newsletters within older adult living communities, and word-of-mouth. Eligible participants were ≥ 60 years-of-age, considered low income according to the 2019 United States Census guidelines via self-report (22), living independently, and completed all physical function assessments. Those with medical implants (i.e., pacemakers, metal implants) and those living in care facilities (i.e., assisted living, skilled nursing, etc.) were excluded from participation.

### 2.2 Bioelectrical impedance analysis

**FIGURE 2:**
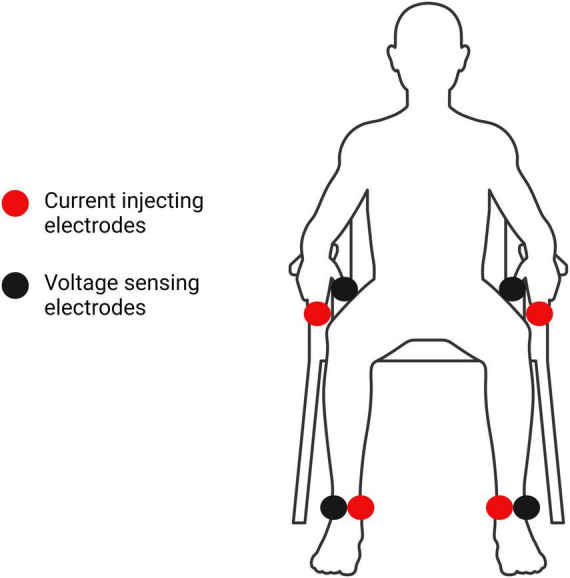
Locations of the InBody s10 current injecting and voltage sensing touch-type electrodes in the seated position (52). Electrodes were placed on the left and right thumbs, middle fingers, and ankles, inferior to the malleoli.

Participants’ height and weight were assessed without shoes using a digital physician scale and stadiometer (Health-O-Meter™, Model 402KL, McCook, IL, United States). Body Mass Index (BMI) was calculated as kg/m^2^. BIA assessments were then conducted using an InBody s10 direct segmental multi-frequency BIA device (Biospace, Seoul, South Korea). The InBody s10 self-calibrated upon each start-up prior to data collection, and all BIA assessments were conducted by trained research assistants. Prior to testing, participants were instructed to fast for at least 3 h, abstain from caffeine for at least 12 h, and avoid strenuous physical activity/exercise as well as alcohol for at least 24 h. Participants sat in a sturdy chair with their socks, shoes, and metal jewelry removed. An InBody Tissue (Biospace, Seoul, South Korea) was used to prepare the skin, and touch-type electrodes were placed on both middle fingers, thumbs, and ankles, as shown in Figure 2. Participants remained seated and silent during the BIA assessment, which lasted approximately 90 s. The InBody s10 has demonstrated good test-retest reliability among older adults with an ICC of 0.82 and 95% confidence interval of 0.71–0.90 (23).

From BIA, we extracted body fat percentage derived from the InBody s10’s proprietary estimation equation as a demographic variable and not a main outcome variable. We extracted bioimpedance variables at 50 kHz as our main outcome variables, specifically Z, R, Xc, R/Height, Xc/Height, PhA, and LMI.

### 2.3 Frailty assessment

Frailty was determined using the 5-item FRAIL questionnaire, which assesses fatigue, resistance, ambulation, illness, and loss of weight (24). The FRAIL questionnaire is a quick, standard assessment of frailty that scores each item as 0 or 1 based on the absence or presence of each frailty characteristic (i.e., item), respectively (24, 25), and the score of each item is summed to determine the total score. Based on their overall score, participants were classified as robust (score = 0), pre-frail (score = 1–2), or frail (score = 3–5). The questionnaire was administered in-person on paper and has previously been validated with diverse populations (24, 25).

### 2.4 Physical function assessments

Physical function was assessed as postural sway (PS), handgrip strength (HGS), Timed-Up-and-Go (TUG), Sit-to-Stand (STS), and Short Physical Performance Battery (SPPB) performance. All tests were administered by trained research assistants. Participants rested between tests at volition.

PS was assessed using a portable Balance Tracking System (BTrackS) platform (Balance Tracking Systems, San Diego, CA, United States). The BTrackS measured center-of-pressure postural sway path length during a 20 s static stance with feet placed approximately hip-width apart on the pre-marked balance plate, hands placed on the hips, eyes closed, and a sturdy piece of furniture or walker placed within the participant’s reach to mitigate falling risk. Each participant completed a 20-s familiarization trial that did not count toward their final score, immediately followed by three 20-s trials that were averaged to calculate their final score, in centimeters. The BTrackS Balance System has demonstrated good test-retest reliability among community-dwelling older adults in assessing center-of-pressure postural sway path length in the eyes-closed condition with an ICC_2, 1_ of 0.83 and a 95% confidence interval of 0.71–0.90 (26).

HGS was measured as maximal isometric force in kilograms (kg) with a JAMAR PLUS digital hand dynamometer (JLW Instruments, Chicago, IL, United States). Participants sat on a sturdy chair with back support and with their feet flat on the floor, elbow bent at 90°, and dynamometer in their hand. The dynamometer was adjusted to allow for a flat second metacarpal and 90° bend at the knuckles. Participants squeezed the dynamometer as hard as possible for 3–5 s across three consecutive trials, with 30-s rest intervals between each trial. The maximum recorded value for each hand was averaged and included for analysis. Among community-dwelling older adults, JAMAR hand dynamometry has demonstrated excellent test-retest reliability when assessing maximal force, with an ICC of 0.97 and 0.96 for the left and right hands, respectively (27).

The TUG was a 3-m normal speed walking test starting with participants seated in a sturdy chair. Upon prompting, the participant would stand up, walk at their normal pace in a straight line to a taped marking on the floor 3 m away, turn around, walk back to the chair at their normal pace, and sit down. Time was recorded in seconds beginning at the research assistant’s prompting and ending when the participant sat back down in the chair. The duration of the TUG was the total score, in seconds. Participants were familiarized with the TUG immediately before the assessment and completed one trial. Previous research has demonstrated clinical utility of the TUG in distinguishing between low- and high-physically functioning older adults (28, 29).

The STS test required participants to stand up from a chair as many times as possible within 30 s. During the test, participants sat in the middle of a sturdy chair with wrists crossed and hands resting on opposite shoulders. A trained research assistant counted each repetition out loud. A repetition was only counted if the participant stood fully erect and sat down entirely. The STS has demonstrated good test-retest reliability with an ICC of 0.85 and a 95% confidence interval of 0.69–0.93 (30).

A phone application (SPPB Guide, Novartis Pharmaceuticals Corporation, Basel, Switzerland) was used for the SPPB to provide standardized prompts, timers, and scoring for each assessment. The SPPB includes assessments of gait speed, lower body power via chair stands, and balance tests. Gait speed is the time, in seconds, required to walk 4 meters at a usual walking pace beginning in the standing position. For the chair stand test, participants started seated and completed five sit-to-stand repetitions as quickly as they could with their arms crossed over the chest, with a repetition only counting if they stood completely erect. The time needed to complete five consecutive chair stands was recorded. The balance tests included maintaining a static position with feet side-by-side, semi-tandem, and tandem for 10 s each; because the duration is fixed, participants were only scored on their ability to hold each position for the full duration. Scores for gait speed, chair stand, and balance tests were summed according to previous literature to provide the total score for the SPPB (31). Scores ranged from 0 to 12, with a higher score representing greater physical function (31).

### 2.5 Statistical analyses

All data were stored in a REDCap database managed by the University of Central Florida (32, 33). All statistical analyses were completed using jamovi version 2.5.6 (34, 35). The main outcome variables were Z, R, Xc, PhA, LMI, R/Height, Xc/Height, HGS, PS, TUG, and SPPB values. Age, height, and body fat percentage were also included in the between group analyses only as demographic variables. A Kolmogorov-Smirnov test confirmed that HGS, PS, TUG, and SPPB data were non-normally distributed, requiring non-parametric tests for those variables. Levene’s test confirmed unequal variances for PS, TUG, and SPPB data; however, results did not differ after accounting for heteroscedasticity. Relationships between variables were assessed using Pearson (r) or Spearman rho correlation coefficients (ρ), and bioimpedance indices and physical function were compared between FRAIL categories using a one-way ANOVA with Tukey *post-hoc* pairwise comparisons or a Kruskal-Wallis H test with Dwass-Steel-Critchlow-Flinger (DSCF) *post-hoc* pairwise comparisons (36). Observed statistical power for the one-way ANOVA and the Kruskal-Wallis H test was calculated using G*Power version 3.1.9.6 (37). Data are presented as mean ± standard deviation unless otherwise indicated. The threshold for statistical significance was set at *p* < 0.05.

## 3 Results

After screening, 208 participants were included in the analyses from 274 recruited. Table 1 provides demographic characteristics. Table 2 provides correlation coefficients between bioimpedance indices and physical function variables as well as FRAIL overall scores within a heat map. Age was well correlated with LMI (ρ = −0.30, *p* < 0.001), PhA (*r* = −0.41, *p* < 0.001), and Xc/Height (*r* = −0.31, *p* < 0.001). Accounting for this, partial correlations controlling for age (i.e., with age as a covariate) are shown in Table 3, with changes in significance highlighted.

**TABLE 1:** Participant characteristics (*N* = 208).

	All	Robust (*n* = 80)	Pre-frail (*n* = 102)	Frail (*n* = 26)
Variable	Mean ± SD or *n* (%)	Mean ± SD or *n* (%)	Mean ± SD or *n* (%)	Mean ± SD or *n* (%)
Age (years)	74.2 ± 6.9	73.3 ± 6.2	75.0 ± 7.1	74.1 ± 8.0
Height (cm)	159 ± 8.0	159 ± 8.1	160 ± 8.1	157 ± 6.9
BMI (kg/m^2^)	30.4 ± 6.4	28.9 ± 5.7	31.1 ± 6.4	32.5 ± 7.8
Body fat percentage (%)	38.9 ± 9.7	37.1 ± 9.6	39.6 ± 9.5	41.1 ± 10.2
Phase angle (°)	5.5 ± 0.9	5.6 ± 0.9	5.4 ± 0.9	5.3 ± 0.9
LMI (°⋅cm/Ω)	1.66 ± 0.49	1.67 ± 0.53	1.67 ± 0.47	1.63 ± 0.45
Impedance (Ω)	545 ± 86.0	561 ± 82.2	536 ± 88.2	529 ± 83.5
Reactance (Ω)	51.6 ± 10.8	54.8 ± 10.0	49.9 ± 11.3	48.2 ± 8.4
Resistance (Ω)	542 ± 85.7	558 ± 82.1	533 ± 87.9	527 ± 83.5
Xc/height (Ω/m)	32.4 ± 6.8	34.5 ± 6.5	31.3 ± 7.1	30.7 ± 5.3
R/height (Ω/m)	342 ± 59.7	352 ± 59.0	335 ± 59.7	337 ± 59.2
Sex	M: 25 (12.0%)	M: 9 (11.3%)	M: 16 (15.7%)	M: 0 (0%)
	F: 183 (88.0%)	F: 71 (88.7%)	F: 86 (84.3%)	F: 26 (100%)
Race/ethnicity	AA: 85 (40.9%)	AA: 27 (33.8%)	AA: 46 (45.1%)	AA: 12 (46.1%)
	A: 17 (8.2%)	A: 5 (6.3%)	A: 8 (7.8%)	A: 4 (15.4%)
	H: 68 (32.7%)	H: 29 (36.2%)	H: 35 (34.3%)	H: 4 (15.4%)
	NHW: 34 (16.3%)	NHW: 17 (21.2%)	NHW: 13 (12.8%)	NHW: 4 (15.4%)
	Other: 4 (1.9%)	Other: 2 (2.5%)	Other: 0 (0%)	Other: 2 (7.7%)
HGS (kg)	19.8 ± 7.5	19.9 ± 6.1	20.6 ± 8.3	15.9 ± 6.9
PS (cm)	32.8 ± 18.4	30.0 ± 16.0	34.0 ± 20.2	36.9 ± 17.3
TUG (s)	10.50 ± 6.76	9.75 ± 7.81	9.81 ± 4.77	15.6 ± 7.84
SPPB	8.8 ± 2.3	9.6 ± 1.7	8.7 ± 2.4	6.8 ± 2.6
STS	11.4 ± 5.1	12.9 ± 4.8	11.4 ± 4.6	6.5 ± 4.8

Xc, Reactance; R, Resistance; SD, Standard deviation; BMI, Body Mass Index; LMI, Levi’s Muscle Index; HGS, Handgrip strength; PS, Postural sway; TUG, Timed-Up-and-Go; SPPB, Short Physical Performance Battery; STS, Sit-to-Stand, measured in repetitions; AA, African American; A, Asian; H, Hispanic; NHW, Non-Hispanic White.

**TABLE 2:** Correlation heat map between bioimpedance indices and physical function (*N* = 208).

Variable	Levi’s Muscle Index		Phase angle		Xc/height		R/height	
	ρ	*p*-value	ρ	*p*-value	ρ	*p*-value	ρ	*p*-value
Handgrip strength	0.46	<0.001	0.45	<0.001	0.12	0.09	−0.28	<0.001
Postural sway	0.08	0.28	−0.13	0.07	−0.26	<0.001	−0.23	<0.001
Timed-up-and-go	−0.05	0.51	−0.17	0.02	−0.21	0.002	−0.08	0.24
SPPB	0.17	0.01	0.32	<0.001	0.23	<0.001	0.03	0.70
	***r***	***p*-value**	***r***	***p*-value**	***r***	***p*-value**	***r***	***p*-value**
FRAIL score	−0.05	0.49	−0.18	0.01	−0.24	<0.001	−0.10	0.13
Levi’s Muscle Index	–	–	0.76	<0.001	−0.07	0.31	−0.77	<0.001
Sit-to-stand	0.05	0.45	0.17	0.01	0.20	0.004	0.08	0.27
Phase angle	0.76	<0.001	–	–	0.58	<0.001	−0.25	<0.001
Xc/height	−0.07	0.31	0.58	<0.001	–	–	0.64	<0.001

ρ, Spearman’s rho correlation coefficient; *r*, Pearson correlation coefficient; SPPB, Short Physical Performance Battery; Xc, Reactance; R, Resistance. Correlation coefficients were interpreted as small (≤ 0.30; yellow), medium (0.30–0.4.9; pink), and large (≥ 0.50; orange). The threshold for statistical significance was *p* < 0.05.

**TABLE 3:** Partial correlation heat map between bioimpedance indices and physical function with age as a covariate (*N* = 208).

Variable	Levi’s Muscle Index		Phase angle		Xc/height		R/height	
	ρ	*p*-value	ρ	*p*-value	ρ	*p*-value	ρ	*p*-value
Handgrip strength	0.40	<0.001	0.38	<0.001	0.03	0.68	−0.28	<0.001
Postural sway	0.14	0.04	−0.05	0.51	−0.21	0.002	−0.24	<0.001
Timed-up-and-go	−0.01	0.91	−0.12	0.08	−0.18	0.01	−0.09	0.20
SPPB	0.10	0.17	0.23	<0.001	0.16	0.02	−0.24	<0.001
	***r***	***p*-value**	***r***	***p*-value**	***r***	***p*-value**	***r***	***p*-value**
FRAIL score	−0.03	0.64	−0.17	0.02	−0.23	0.001	−0.11	0.13
Levi’s Muscle Index	–	–	0.75	<0.001	−0.16	0.02	−0.78	<0.001
Sit-to-stand	0.02	0.75	0.13	0.06	0.17	0.01	0.08	0.24
Phase angle	0.75	<0.001	–	–	0.52	<0.001	−0.25	<0.001
Xc/height	−0.16	0.02	0.52	<0.001	–	–	0.68	<0.001

ρ, Spearman’s rho correlation coefficient; *r*, Pearson correlation coefficient; SPPB, Short Physical Performance Battery; Xc, Reactance; R, Resistance. The threshold for statistical significance was *p* < 0.05. Correlation coefficients were interpreted as small (≤ 0.30), medium (0.30–0.4.9), and large (≥ 0.50). Partial correlations that became statistically significant after including age as a covariate are highlighted in green. Partial correlations that became statistically non-significant after including age as a covariate are highlighted in orange.

The one-way ANOVA revealed significant differences between FRAIL categories for Z, Xc, R, and Xc/Height (Table 4). Tukey pairwise comparisons showed significant differences between robust and pre-frail participants in Xc (*t* = 3.13, *p* = 0.01, *d* = 0.55) and Xc/Height (*t* = 3.36, *p* = 0.003, *d* = 0.59). There were significant differences between robust and frail participants in Xc (*t* = 2.50, *p* = 0.04, *d* = 0.68), and Xc/Height (*t* = 2.54, *p* = 0.03, *d* = 0.69), No other Tukey pairwise comparisons were significant. Despite significant omnibus effects, there were no significant pairwise comparisons for Z nor R. Results for PhA, Xc/Height, and LMI did not change after controlling for age with an ANCOVA.

**TABLE 4:** One-way comparisons between FRAIL categories (*N* = 208).

Variable	*F*_(2, 205)_	*p*-value	η_*p*_^2^	Observed power
Age	1.35	0.26	0.01	0.23
Height	1.28	0.28	0.01	0.23
Body fat percentage	2.33	0.10	0.02	0.43
Impedance	2.47	0.09	0.02	0.43
Reactance	6.45	0.002	0.06	0.91
Resistance	2.41	0.09	0.02	0.43
Xc/height	6.39	0.002	0.06	0.91
R/height	2.05	0.13	0.02	0.43
Levi’s Muscle Index	0.05	0.95	0.001	0.07
Phase angle	2.63	0.07	0.03	0.61
Sit-to-stand	17.8	<0.001	0.15	0.99
	**X^2^_(2)_**	***p*-value**	**ε^2^**	
SPPB	22.96	<0.001	0.11	0.06
Handgrip strength	13.74	0.001	0.07	0.06
Postural sway	3.29	0.19	0.02	0.05
Timed-up-and-go	20.45	<0.001	0.10	0.06

η_*p*_^2^, partial eta squared effect size for a one-way ANOVA test, where 0.01, 0.09, and 0.25 represent small, medium, and large effect sizes, respectively.

ε^2^, epsilon squared effect size for a Kruskal-Wallis H test, where 0.01, 0.06, and 0.14 represent small, medium, and large effect sizes, respectively. Xc, Reactance; R, Resistance; SPPB, Short Physical Performance Battery. The threshold for statistical significance was *p* < 0.05.

The Kruskal-Wallis H test revealed significant differences between FRAIL categories for SPPB, TUG, and HGS performance (Table 4). DSCF pairwise comparisons showed significant differences between robust and frail participants in SPPB (W = −6.81, *p* < 0.001), TUG (W = 6.10, *p* < 0.001), and HGS performance (W = −5.02, *p* = 0.01). There were significant differences between pre-frail and frail participants in SPPB (W = −4.40, *p* = 0.005), TUG (W = 5.70, *p* < 0.001), and HGS performance (W = −4.86, *p* = 0.002). Robust and pre-frail participants did not differ in TUG (W = 1.44, *p* = 0.57) nor HGS performance (W = −0.21, *p* = 0.99), but they did significantly differ in SPPB performance (W = −3.44, *p* = 0.04). No other DSCF pairwise comparisons were statistically significant.

## 4 Discussion

The primary purpose of this study was to examine the relationships between bioimpedance indices, physical function, and frailty among older adults. The results partially supported our hypothesis; from BIA, only Xc and Xc/Height significantly differed between frailty classifications (Table 4). Regarding physical function assessments, SPPB, TUG, and HGS performance differed between frailty classifications as well (Table 4). Xc/Height was significantly correlated with FRAIL scores and all physical function assessments except HGS. R/Height was significantly correlated with all physical function assessments except TUG and was not well associated with FRAIL scores. Of the physical function assessments, LMI was only significantly associated with HGS and SPPB, while PhA was significantly associated with TUG, HGS, SPPB, and STS performance. However, Xc/Height was the only bioimpedance index to maintain significant relationships with both physical function assessments and FRAIL scores after controlling for age, suggesting that other bioimpedance indices may not be strong and reliable indicators of physical function outside of HGS.

To the best of our knowledge, the present study is the first study using LMI with an older adult population. LMI has previously been used to distinguish training level (i.e., elite vs. high vs. medium) between Italian football (soccer) athletes (15), with significant differences between levels. While training levels between athletes are not synonymous with frailty classifications, they both represent physical capacity. However, the lack of differences between frailty categories in the present study despite significant differences in physical function suggests that LMI may not reflect physical capacity among older adults. This is despite LMI incorporating PhA into its calculation, which was significantly associated with multiple aspects of physical function in the present study as well as previous work from our lab with a similar sample of older adults (5).

While LMI has not been widely applied among older adults, Xc/Height and R/Height are commonly used with older adult populations in bioelectrical impedance vector analysis to graph and categorize individuals as “lean,” “athletic,” “obese,” or “cachectic” (38). Xc/Height and R/Height are valid methods for assessing malnutrition and cardiometabolic disorders (13, 39). However, previous research examining relationships between bioimpedance variables and frailty have largely ignored Xc/Height and R/Height (11, 40, 41). Aging does not appear to moderate the relationships Xc/Height and R/Height have with physical function measures. Despite this, Xc/Height was the only bioimpedance index to differ between frailty classifications, demonstrating an ability to distinguish between robust and pre-frail older adults, which no physical function assessment was able to do except for the SPPB. Xc is representative of the capacitive reactance of cell membranes (i.e., the ability for cell membranes to hold onto electrical currents and discharge them) and is independent of R (8, 9), so the observed ability for Xc/Height to differentiate between frailty categories may suggest changes in capacitive reactance but not R occurring with frailty, although future research is needed to confirm this theory. R/Height, Z, and R did not significantly differ between frailty classifications. R, which comprises the majority of Z (42), is closely related to adiposity (i.e., body fat percentage; (43)), which did not differ between frailty classifications and may explain why R/Height did not differ between frailty classifications. Nonetheless, both Xc/height and R/Height may be clinically relevant indicators of physical function, yet only Xc/height appears to be a clinically relevant indicator of frailty. Researchers and clinicians should be cautious when attempting to infer physical function or frailty status from R/Height, LMI, and PhA, despite the common clinical use of PhA (9–12). Given the SPPB’s ability to differentiate between all three frailty classifications in the present study and previous research (44), pairing bioimpedance indices with the SPPB may provide a more robust screening for frailty than a single bioimpedance index alone.

Compared to the lack of previous research on Xc/Height, R/Height, and frailty, the relationship between PhA and frailty has been examined before with equivocal results (4, 11, 40, 41). While we compared PhA between robust, pre-frail, and frail categories, Araujo et al. combined the pre-frail and frail groups when comparing PhA to robust older adults (4). Like the present study, Arajuo et al. observed no significant differences in PhA between robust and pre-frail/frail older adults (4), although their results contrast Kolodziej et al. who also collapsed the pre-frail and frail groups into one, reporting significant differences in PhA between non-frail and pre-frail/frail older men and women (41). Likewise, Tanaka et al. classified frailty in a binary fashion with the Japanese version of the Cardiovascular Health Study (J-CHS) criteria for determining frailty, which focuses on fatigue, poor grip strength, inactivity, unintentional weight loss, and slow walking speed (11). Similar to Kolodziej et al. and Tanaka et al. reported significant differences in PhA between non-frail and frail older adults (11, 41). Furthermore, Saitoh et al. used the J-CHS with older adults and concluded that low PhA was significantly associated with an increased risk of frailty (40). While the FRAIL questionnaire assesses similar domains as the J-CHS, the assessments and questions within each frailty index differ, potentially leading to unique relationships with bioimpedance indices. This underscores a key issue in establishing indicators of frailty: a lack of standardization. The inconsistent combination of pre-frail and frail groups into a single category may make it difficult to determine the efficacy of indices such as PhA in distinguishing robust and frail older adults. Moreover, although frailty is understood to be multidimensional, subsuming a decrease in physical, psychological, cognitive, and social capacity, there is no uniform definition nor classification of frailty (45). Over 20 different assessments exist to determine frailty (e.g., Fried’s Phenotype, Edmonton Scale, etc.), each with differing approaches to quantifying and categorizing older adults (45). The potential differences in how frailty is assessed may explain the equivocal relationship between PhA and frailty among older adults, as those relationships may only be specific to the measure of frailty used. Future research should examine the relationship between bioimpedance measures (i.e., PhA and LMI) and frailty using multiple established frailty indices. Additionally, standard guidelines for assessing and classifying frailty are needed to improve clinical practice.

Our results should be interpreted cautiously, as we observed low statistical power in several of our between-group comparisons (Table 4) due to sample size and small effect sizes, indicating that further research with larger samples is needed to validate our results. Beyond using a single frailty index, our study had limited representation of male and frail participants. However, previous research has demonstrated a longer life expectancy for women in the United States (46, 47) and a global prevalence of frailty ranging from 4 to 16% (48, 49), compared to the observed prevalence of 12.5% in the present study. This suggests that our sample is representative of a random sample of community-dwelling older adults. Additionally, our results did not differ when analyzed without the male participants, indicating no significant influence of the male sub-sample on the overall observed results from our full sample. While previous studies have had low sample sizes for frail older adults and chose to combine them with pre-frail older adults, our study’s significant differences in physical function and bioimpedance indicate that pre-frail and frail older adults are not homogeneous as a single sample. Our sample of frail older adults also provides novel insights into the applicability of LMI, as previous research with LMI has primarily focused on healthy and athletic populations. Santangelo et al. measured LMI in healthy expeditioners before a 4-day trekking trip in participants that were 29.25 ± 4.16 years of age (18). They reported LMI values of 1.5°⋅cm/Ω for female participants (18), which is lower than the mean 1.63°⋅cm/Ω value reported in the present study for frail older adults. In another study with elite Italian track and field athletes, the female athletes had a mean LMI of 1.9°⋅cm/Ω (17), which is a mean difference of less than 0.3°⋅cm/Ω compared to frail older adults. More research is needed using LMI with non-athletic populations, as the observed LMI values in our study with frail older adults appears to be like that of younger, female, athletic adults from previous research (17, 18). We also included robust physical function assessments, providing novel insights into the relationship between LMI and physical function as a surrogate for muscle quality. More research with recruitment specifically targeting frail older adults may be needed to examine the utility of LMI with older adults further. Furthermore, future studies may be able to utilize electrical impedance myography to assess LMI with a single muscle and compare its assessment of muscle quality with more evidence-based approaches, such as echo intensity via ultrasound (50, 51).

## 5 Conclusion

This study aimed to assess the relationships between LMI, PhA, Xc/Height, R/Height, physical function, and frailty among older adults. Neither LMI nor PhA appear to be indicative of frailty among community-dwelling older adults. Despite including PhA in its calculation, LMI was only significantly correlated with HGS and the SPPB out of all included physical function measures, while PhA was associated with HGS, TUG, STS, and SPPB performance. Xc/Height and the SPPB could distinguish physical function between frailty classifications without age being a moderating factor. Clinicians and researchers may be able to infer frailty status among older adults using Xc/Height and/or SPPB performance. However, more research comparing BIA to other frailty indices/assessments is needed to support the concurrent validity of BIA indices as frailty assessments.

## Data Availability

The raw data supporting the conclusions of this article will be made available by the authors, without undue reservation.
